# The Deep-Time Digital Earth program: data-driven discovery in geosciences

**DOI:** 10.1093/nsr/nwab027

**Published:** 2021-02-11

**Authors:** Chengshan Wang, Robert M Hazen, Qiuming Cheng, Michael H Stephenson, Chenghu Zhou, Peter Fox, Shu-zhong Shen, Roland Oberhänsli, Zengqian Hou, Xiaogang Ma, Zhiqiang Feng, Junxuan Fan, Chao Ma, Xiumian Hu, Bin Luo, Juanle Wang, Craig M Schiffries

**Affiliations:** State Key Laboratory of Biogeology and Environmental Geology, China University of Geosciences, Beijing 100083, China; School of the Earth Science and Resources, China University of Geosciences, Beijing 100083, China; Earth and Planets Laboratory, Carnegie Institution for Science, Washington, DC 20015, USA; State Key Laboratory of Geological Processes and Mineral Resources, China University of Geosciences, Beijing 100083, China; BritishGeological Survey, Nottingham, NG12 5GG, UK; State Key Laboratory of Resources and Environment Information System, Institute of Geographical Science and Natural Resources, Chinese Academy of Sciences, Beijing 100101, China; Tetherless World Constellation, Rensselaer Polytechnic Institute, Troy, NY 12180, USA; School of Earth Sciences and Engineering, Nanjing University, Nanjing 210023, China; Institute of Earth and Environmental Sciences, University of Potsdam, Potsdam 14476, Germany; Institute of Geology, Chinese Academy of Geological Sciences, Beijing 100037, China; Department of Computer Science, University of Idaho, Moscow, ID 83844, USA; Petroleum Exploration and Production Research Institute, SINOPEC, Beijing 100083, China; School of Earth Sciences and Engineering, Nanjing University, Nanjing 210023, China; Department of Computer Science, University of Idaho, Moscow, ID 83844, USA; School of Earth Sciences and Engineering, Nanjing University, Nanjing 210023, China; State Key Laboratory of Resources and Environment Information System, Institute of Geographical Science and Natural Resources, Chinese Academy of Sciences, Beijing 100101, China; State Key Laboratory of Resources and Environment Information System, Institute of Geographical Science and Natural Resources, Chinese Academy of Sciences, Beijing 100101, China; DDE Center of Excellence (Suzhou), Kunshan 215300, China

**Keywords:** Deep-Time Digital Earth, data-driven discovery, big data, Earth evolution, cyberinfrastructure

## Abstract

Current barriers hindering data-driven discoveries in deep-time Earth (DE) include: substantial volumes of DE data are not digitized; many DE databases do not adhere to FAIR (findable, accessible, interoperable and reusable) principles; we lack a systematic knowledge graph for DE; existing DE databases are geographically heterogeneous; a significant fraction of DE data is not in open-access formats; tailored tools are needed. These challenges motivate the Deep-Time Digital Earth (DDE) program initiated by the International Union of Geological Sciences and developed in cooperation with national geological surveys, professional associations, academic institutions and scientists around the world. DDE’s mission is to build on previous research to develop a systematic DE knowledge graph, a FAIR data infrastructure that links existing databases and makes dark data visible, and tailored tools for DE data, which are universally accessible. DDE aims to harmonize DE data, share global geoscience knowledge and facilitate data-driven discovery in the understanding of Earth's evolution.

## Teaser

Deep-time Digital Earth is to promote data-driven discovery by harmonizing deep-time Earth data and creating ‘Geological Google’.

## OPPORTUNITIES FOR ABDUCTIVE, DEEP-TIME, DATA-DRIVEN DISCOVERY

Humans have long explored three big scientific questions: the evolution of the universe, the evolution of Earth and the evolution of life. Geoscientists have embraced the mission of elucidating the evolution of Earth and life, which are preserved in the information-rich but incomplete geological record that spans more than 4.5 billion years of Earth history. Delving into Earth's deep-time history helps geoscientists decipher mechanisms and rates of Earth's evolution, unravel the rates and mechanisms of climate change, locate natural resources and envision the future of Earth.

Two common approaches have been widely employed for studying Earth's history. *Deductive reasoning* begins with a general premise that is asserted to be true (e.g. the convergence of two lithospheric plates results in the formation of mountains), and then draws specific inferences from that generalization that must also be true (i.e. the Appalachian mountains formed because the European and North American plates converged). By contrast, *inductive reasoning* relies on observations of particular instances of a generalization (e.g. the Alps, the Himalayas and the Appalachian mountains all formed at convergent margins), which then lead to predictions of additional examples of the generalization, or to the generalization itself (i.e. the convergence of two plates results in the formation of mountains).

In contrast to deduction and induction, *abduction* is derived from accumulation and analysis of large amounts of reliable data, independently of a premise or generalization [1]. Abductive discovery is predicated on the conviction that large data resources, especially multi-dimensional data that are not easily visualized, hold patterns that are not easily discovered with deductive or inductive protocols. Mathematical analysis of data leads to discovery of previously hidden patterns, which then points to new generalizations. Abduction thus has the potential to generate transformative discoveries in science, such as Lyell's recognition of gradual geological change through Earth's deep time [2] and Charles Darwin's elucidation of evolution by natural selection [3]. Those discoveries required the inspired integration of numerous lines of evidence to see previously hidden patterns in nature.

With the accumulation of enormous volumes of deep-time Earth data, we are poised to transform research in deep-time Earth science through data-driven abductive discovery. Deep-time Earth data are produced in heterogeneous formats and media—including text, tables, figures, maps, images and videos—and distributed in disparate literature and databases, which need integration and harmonization to achieve broader scientific ambitions. To this end, several thematic data facilities have been constructed (Table 1), such as the Paleobiology Database (PBDB, paleontology, https://paleobiodb.org), Macrostrat (sedimentology and stratigraphy, https://macrostrat.org/), EarthChem (geochemistry, geochronology and petrology, https://www.earthchem.org/) and RRUFF (mineralogy, http://rruff.info/).

**Table 1.** Selected databases and portals currently in use or in development by DDE. GBDB and OneStratigraphy are databases being developed by DDE, while the rest are independent data systems used by DDE.

Database/portal	Scope	URL and references
EarthChem	Access to global geochemical and petrological data syntheses (PetDB, EarthChem Portal, LEPR, traceDs); EarthChem Library publishes and archives geochemical, petrological and mineralogical data as a trusted repository recommended by publishers	earthchem.org[12,13]
Geobiodiversity Database (GBDB)	Integrated system for the management and analysis of section-based stratigraphic and paleontological information	geobiodiversity.com[14,15]
GeoDeepDive and	Digital libraries and cyberinfrastructure facilitating the discovery and utilization of geologic data and knowledge in published documents	geodeepdive.org
PaleoDeepDive		[16–18]
Macrostrat	Collaborative platform for the aggregation and distribution of geological data relevant to the spatial and temporal distribution of sedimentary, igneous and metamorphic rocks as well as data extracted from them	macrostrat.org[17,19]
Mindat	World's largest open database of minerals, rocks, meteorites and the localities they come from	mindat.org[20]
OneGeology Portal	Geologic map data and relevant geoscience data worldwide at scales ≥1 : 1 million	portal.onegeology.org[21]
OneStratigraphy Database	Platform designed for sharing and using stratigraphic data, including integration, management, visualization and analytics of stratigraphic data	onestratigraphy.ddeworld.org[15]
Paleobiology Database (PBDB)	Global, collection-based occurrence and taxonomic data for organisms of all geological ages, as well as data services to allow easy access to data for independent development of analytical tools, visualization software and applications	paleobiodb.org[22]
PANGAEA	Data publisher for Earth and environmental science; open-access library aimed at archiving, publishing and distributing georeferenced data from Earth system research	pangaea.de
RRUFF	High-quality spectral data—including X-ray diffraction, Raman spectra and electron microprobe analyses—from well-characterized minerals	rruff.info[23]
PALEOMAP	Plate tectonic reconstruction during the past 1100 million years	scotese.com[24]
GPlates	An open-source software that offers a novel combination of interactive plate-tectonic reconstructions, geographic information system functionality and raster data visualization	gplates.org[25]

However, three issues must be resolved to facilitate abductive discovery utilizing deep-time databases. First, many relevant geodata resources are not in compliance with FAIR (findable, accessible, interoperable, and reusable) principles for scientific data management and stewardship [4,5]. Second, concepts and terminologies used in databases are not well-defined, thus the same term may have different meanings across databases. Without standardized terminology and definitions of concepts, it is difficult to achieve data interoperability and reusability. Third, databases are highly heterogeneous in terms of geographic regions, spatial and temporal resolution, coverage of geological themes, limitation of data availability, formats, languages and metadata. For example, because most data in popular databases are from the English literature, the diversity, density and richness of these databases are generally low with respect to non-native English-speaking countries, which are nevertheless of critical importance in studying Earth's evolution. Due to the complex evolution of Earth and interactions among multiple spheres (e.g. lithosphere, hydrosphere, biosphere and atmosphere) in Earth systems, it is difficult to see the whole picture of Earth's evolution from separated thematic views, each with limited scope.

Big data and artificial intelligence are creating opportunities for resolving these issues [6–10]. To explore Earth's evolution efficiently and effectively through deep-time big data, we need FAIR, synthetic and comprehensive databases across all fields of deep-time Earth science, coupled with tailored computation methods. This goal motivates the Deep-Time Digital Earth program (DDE; [11]), which is the first ‘big science program’ initiated by the International Union of Geological Sciences (IUGS) and developed in cooperation with national geological surveys, professional associations, academic institutions and scientists around the world. The main objective of DDE is to facilitate deep-time, data-driven discoveries through international and interdisciplinary collaborations. DDE aims to provide an open platform for linking existing deep-time Earth data and integrating geological data that users can interrogate by specifying time, space and subject (i.e. a ‘Geological Google’) and for processing data for knowledge discovery using a knowledge engine (Deep-Time Earth Engine) that provides computing power, models, methods and algorithms. DDE can help scientists with time-consuming data cleansing and processing so they can focus on research topics and discoveries.

## MISSION AND VISION

**Figure 1.**
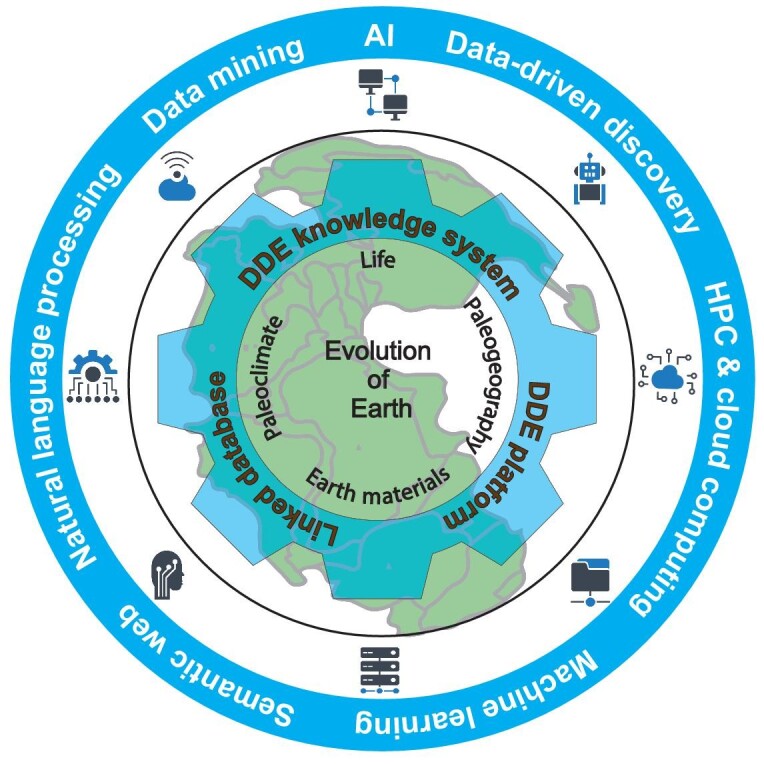
Schematic diagram of DDE system. DDE aims to harmonize deep-time Earth data based on a knowledge system in order to investigate the evolution of Earth, including life, Earth materials, geography and climate. Integrated methods include artificial intelligence, high performance computing, cloud computing, semantic web, natural language processing and other methods.

DDE aims to link and harmonize global deep-time Earth data and share global geoscience knowledge with the goal of stimulating data-driven discoveries in the study of Earth's evolution through deep time. Understanding Earth's past is essential for understanding Earth's present and future. Earth's evolution involves four major themes: life on Earth, Earth materials (such as minerals, rocks, sediments and fluids), geography and climate (Fig. 1; Table 2). Data science and artificial intelligence may provide new techniques for accelerating data-driven discovery in exploratory studies of Earth evolution.

**Table 2.** Selected data-driven scientific goals of DDE.

Topic	Data-driven scientific goals	References
Evolution of life and biodiversity	Understand patterns of global biodiversity through deep time, including high-resolution timing of biological extinction and diversification events, and relationships between environmental changes and biodiversity fluctuations	[15,26,27]
Evolution of Earth materials	Develop novel evolutionary system of Earth materials including minerals, rocks, sediments and fluids. We hypothesize that life greatly influenced the nature and distribution of crustal minerals. Comprehensive and reliable deep-time Earth materials data resources will facilitate the testing of this hypothesis	[20,28–30]
Evolution of geography	Integrate plate tectonic reconstructions with other deep-time Earth databases to facilitate data-driven discoveries in paleobiology, paleoceanography, paleoclimate, geodynamics, tectonics, orogenesis, geochemical cycles, basin evolution, energy and mineral resource exploration, and other topics	[25,31–34]
Evolution of climate	Quantify changes in global climate and atmospheric composition through deep time	[35]

### Evolution of life

Our understanding of the evolution of life on Earth is being propelled by data-driven discoveries. For example, supercomputer and big data analysis applied in paleontology has helped to create a high-resolution profile of Paleozoic biodiversity [15], which reveals, for example, mass extinctions and diversification of early complex life—events that were previously unrecognized [22]. Likewise, network analysis of Ediacaran fauna provides evidence for pulsed extinctions of early complex life [27]. To further facilitate these types of studies, DDE seeks to link and expand existing deep-time paleontological and stratigraphic databases, such as OneStratigraphy (onestratigraphy.ddeworld.org), Macrostrat (macrostrat.org), Paleobiology Database (paleobiodb.org) and PANGAEA (pangaea.de), and integrate them with plate tectonic reconstructions, deep learning and other tools that will accelerate data-driven discovery.

### Evolution of Earth materials

Earth materials, such as minerals, rocks, sediments and fluids, represent time capsules that contain enormous amounts of information about Earth history [20,36]. These materials also include thousands of meteorites that come from other astronomical bodies from outer space—samples that provide rich information about the early solar system. DDE plans to link the existing databases (e.g. mindat.org, rruff.info, earthchem.org) and expand the mineralogical and petrological data, as well as geochemical and geophysical data, for Earth materials, with an aim to mitigate existing biases and increase spatiotemporal coverage and resolution in materials used in data-driven discoveries. These data-driven discoveries in the evolution of Earth materials include, but are not limited to, the rapidly emerging fields of mineral evolution and mineral ecology [36,37], secular lithospheric evolution indicated by igneous rocks [28], plate tectonics inferred from minerals and igneous rocks [29], and sediment cycling [30]. For example, we will test the hypothesis that more than two-thirds of mineral species on Earth are biologically mediated, and thus would not occur on a non-living world. Comprehensive data resources on the ages and distribution of more than 5600 known mineral species would facilitate the effort to understand the deep connections between the geosphere and biosphere.

### Evolution of geography

The evolution of paleogeography is key to understanding Earth history and predicting and assessing occurrences of mineral and energy resources, as well as geohazards. DDE intends to harmonize differences and uncertainties between different open-source plate tectonic reconstructions, such as the PALEOMAP Project [24] and GPlates [25]. DDE also plans to connect other deep-time Earth databases to paleogeographic reconstructions, which can facilitate scientific discoveries based on large amounts of temporal and spatial data from deep-time Earth. For example, integration of paleogeographic maps of carbonate platforms with plate tectonic reconstructions constrains the influence of carbonate platform interactions with subduction zone volcanism on paleoatmospheric CO_2_ since the Devonian [33]. Integrating plate tectonic reconstructions with other deep-time Earth databases [31] has applications to paleobiology [32], paleoceanography, paleoclimate [31], geodynamics [34], tectonics, orogenesis, geochemical cycles, basin evolution, energy and mineral resource exploration, and other topics.

### Evolution of climate

DDE also plans to investigate Earth's paleoclimates (climate in deep time). Climate has varied widely in deep time (hothouse climate to snowball Earth). Shorter-term (thousands of years) and longer-term (millions of years) climatic cycles are also preserved in paleoclimate. These unique climatic variations are difficult to study solely based on recent climate records and climate modeling. Studies of paleoclimate aid our understanding of how Earth and life interact to produce climate extremes and to forecast future climate variations (reading past climate to inform future climate change, [38]). In this regard, DDE aims to reconstruct Earth's paleoclimate and paleoatmosphere history based on various mineralogical and geochemical indexes, as preserved in Earth materials. For example, deep-time geological data related to the Cretaceous hothouse climate could be used to understand the evolution of climate extremes in more recent times.

### Natural resources and applied science

DDE has the potential to address applied geological problems in such areas as energy, mineral resources and environmental protection, by working with geological surveys, government agencies, private companies and civil societies to improve energy and resource security, tackle modern day climate change and contribute to the UN’s Sustainable Development Goals. These objectives can be achieved by integrating databases to answer pressing applied geoscience questions, such as discovering porphyry copper mineral deposits, especially by relating such deposits to deep-time tectonic plate motion and crustal and slab subduction [39,40]. Applying data analysis tools to linked databases and models can provide insights into ore deposits that are not possible through simple analysis of single or pairs of databases. DDE will link georeferenced databases and models of this type so that they can be used more efficiently.

The spheres of the Earth system—the geosphere, hydrosphere, atmosphere and biosphere—interact with each other. Associations among these spheres can be explored by linking big data in deep time. The hidden relationships and mutual influences among spheres can be revealed by methods of data-driven discovery. For example, studying the evolution of Earth's sediments based on sedimentary rock databases could help to decode Earth's dynamic and varied crustal evolution [41]. As another example, geochemical and mineralogical data mining has demonstrated that the assembly of the Rodinian supercontinent ∼1 billion years ago was unique [29]. Data-driven approaches applied to global zircon age data, integrated with variations of mantle temperature, supercontinent formation, continent growth rate and more, reveal the secular changes of continental evolution, which can be extrapolated to predict the future of plate tectonics [42]. These data-driven approaches have raised new questions about whether extreme events, such as mass extinctions of life, the formation of large igneous provinces and abrupt atmospheric changes, are random, episodic or periodic events. New mathematical and computational approaches of data discovery may help answer these questions. This data-driven approach is a significant change in the way we make scientific discoveries—a complement to more traditional modes of experiments and modeling. Significant progress in data-driven discovery will require the integration of varied Earth data in deep time. This integration will be accelerated by a standard knowledge system of Earth in deep time. Thus, the goal of DDE is to link and harmonize global data from all of Earth's deep-time disciplines, employing FAIR practices, rebuilding the knowledge system based on existing ontologies of deep-time Earth, and sharing the knowledge with scientists, the public and governments. DDE aims to transform geosciences, complementing their traditional inductive and deductive approaches with the data-driven power of abductive discovery in the study of Earth's evolution.

## STRUCTURE OF THE DDE PROGRAM

To achieve its mission and vision, the DDE program has three main components: program management committees, centers of excellence, and working, platform and task groups.

### Program committees

As an international big science program, DDE is operated and managed by leading scientists and data science professionals. It has three management bodies: (i) Governing Council (GC), which consists of representatives of DDE’s founding organizations (Box 1); (ii) Science Committee (SC), which is an advisory body on scientific matters, and conducts thorough evaluation of scientific activities and research proposals; and (iii) Executive Committee (EC), which oversees and coordinates DDE’s day-to-day operations and communications with assistance from the DDE Secretariat Office. The GC provides governance, financial and scientific overview; it makes major policy decisions and reviews the acceptance of new members and new centers for the program. DDE is still in its initiation and development stage. Any institutes or organizations who are willing to share deep-time data and promote data-driven science are welcome to join the program.

**Box 1. DDE founding members.**

1. British Geological Survey (BGS)
2. China Geological Survey (CGS)
3. Geological Survey of Canada (GSC)
4. Korean Institute of Geoscience and Mineral Resources (KIGAM)
5. Russian Geological Research Institute (VSEGEI)
6. American Association of Petroleum Geologists (AAPG)
7. Coordinating Committee for Geoscience Programmes in East and Southeast Asia (CCOP)
8. Commission on Geoscience Information (CGI)
9. Commission for Geological Map of The World (CGMW)
10. Russian Federal Geological Foundation (FBGU)
11. International Association of Geomorphologists (IAG)
12. International Association on the Genesis of Ore Deposits (IAGOD)
13. International Association for Mathematical Geoscience (IAMG)
14. International Association of Sedimentologists (IAS)
15. International Commission on Stratigraphy (ICS)
16. International Lithosphere Program (ILP)
17. International Palaeontological Association (IPA)
18. Geological Survey of India (GSI)

### Research centers

DDE plans to develop three connected research centers of excellence around the world. The first center is being built in Suzhou, China, with financial and infrastructure support from the Suzhou municipal government. The Suzhou center aims to host 100 research scientists and technicians to advance the goals of DDE. The second center of excellence will be developed in the US in collaboration with the Deep-Time Data-Driven Discovery initiative (4D, https://4d.carnegiescience.edu). The main objective of the US-based center of excellence is to harness the growing momentum of collaborative Earth–space–life data-driven research. The third center of excellence under consideration is to be in Europe. These DDE centers of excellence will have four main missions. First, the centers will integrate geoscience and data science. A team of scientists with interdisciplinary expertise in geoscience and data science will be formed to facilitate data-driven discoveries by DDE. This team will support every DDE working and task group and will be backed by professional data scientists, who specialize in data analysis and computing. Second, the centers will provide computational services to DDE working groups and collaborators to analyze data. Third, the centers will host several key laboratories for generating geoscience data, such as geochronological and stable isotope data. Fourth, the centers will provide training and education for professional scientists as well as information for the public.

### Working, platform and task groups

DDE now has three categories of groups: working, platform and task groups (Table 3) that have a mandate to: (i) build knowledge graphs and data infrastructure of deep-time Earth; and (ii) investigate Earth's evolution by employing big data analysis. The working and platform groups are long-term, while the DDE task groups are short-term, ad hoc bodies with specific objectives. These groups are formed by international collaborations and each group has its own group leader. These groups are part of DDE and will be supported long-term and led by DDE for source allocation and work consistency. On the other hand, the program is open to experts from any country to propose working or task groups with well-defined tasks, which will be reviewed by the DDE’s EC and GC. DDE will provide support (e.g. financial) and resources (e.g. computing power) to these ‘open groups’. Consistency between these groups is monitored by DDE’s SC, EC and GC.

**Table 3.** DDE working, platform and task groups.

Groups		Mission
Thematic working groups^a^		Build DDE knowledge graph and standards
		Assess data quality and accuracy
Platform group		Harmonize and clean data
		Create storage and computational platform, such as cloud storage and computing
		Develop DDE application system
Task groups	Regional groups^b^	Harmonize regional geological databases
		Identify and solve regional science and resources problems
	Climate modeling group	Harmonize climate modeling data
	Popular science, education and communications group	Popular science
		Provide data science training to geoscientists and geoscience training to data scientists
		Communicate DDE knowledge and products to non-technical audiences, including the general public

^a^Paleontology, Paleogeography, Paleomagnetism, Sedimentology, Stratigraphy, Metamorphic Rocks, Igneous Rocks, Geochronology, Tectonics, Geophysics, Geothermics, Hydrology, Petroleum Geology.

^b^Central Asia, Southeast Asia, Marginal sea.

## RESEARCH PLAN

**Figure 2.**
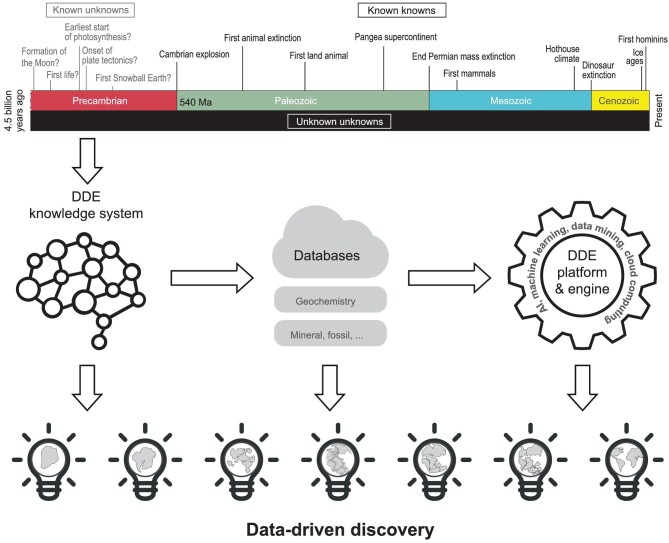
Workflow of data-driven discovery in DDE. Scientific questions in Earth history can be addressed using the knowns and unknowns framework: (i) Known knowns. This category, which is relative to the other two, includes widely accepted and broadly understood events in Earth history, although uncertainties still exist. (ii) Known unknowns. This category includes events that are widely accepted to have happened but key aspects are poorly understood. In many cases, hypotheses about such events can be tested with additional observations, measurements or experiments. (iii) Unknown unknowns. This category includes events that took place in the Earth's history but have not been discovered. Through its knowledge system and platform, DDE aims to harmonize deep-time Earth data and promote data-driven discovery in these unknowns, especially unknown unknowns in Earth history. Note: the time scales of Precambrian and Phanerozoic differ in scale.

In order to achieve its mission and goals, DDE will build on existing deep-time Earth knowledge systems and develop an open platform (Fig. 2). A deep-time Earth knowledge system consists of the basic definitions and relationships among concepts in deep-time Earth, which are necessary for harmonizing deep-time Earth data and developing a knowledge engine for supporting abductive exploration of Earth's evolution.

**Figure 3.**
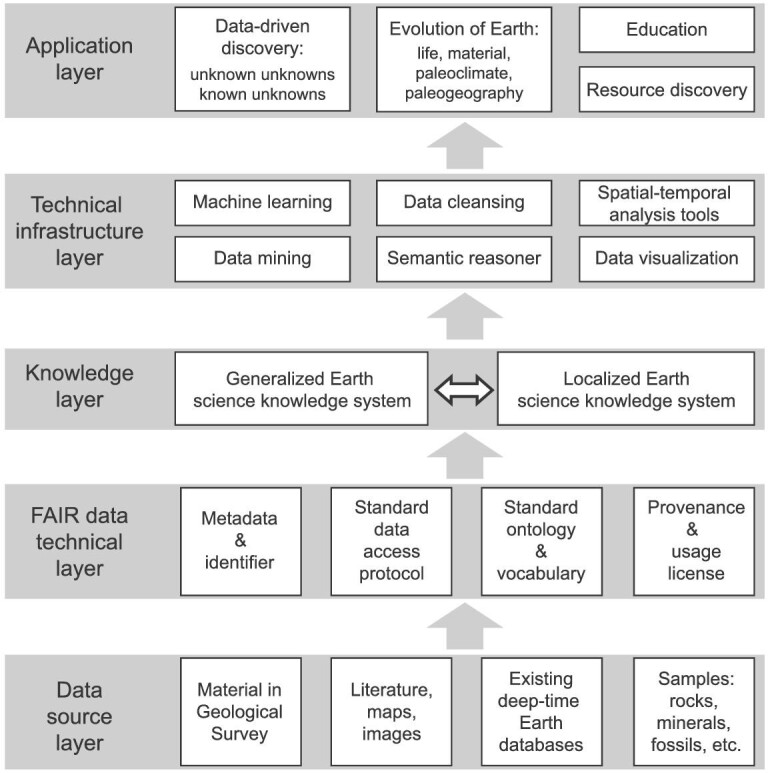
Structure of the DDE technology system. From bottom to top: data source integration, data cleansing and sharing, knowledge base construction, technical infrastructure configuration, and applications for scientific discovery.

The first step in DDE’s research plan is to build on existing deep-time Earth knowledge systems. Earth scientists have developed various indices to represent and interpret Earth's evolution. These indices can be coded as graphs representing an Earth science knowledge system. The system includes the definitions and associations of the concepts and terminologies in deep-time Earth science. These computer-coded graphs of knowledge systems can support applications of artificial intelligence. Establishing this knowledge system is a necessary step for DDE’s semantically cohesive data infrastructure. It helps ensure the data (linked and expanded) are FAIR (Fig. 3). Pioneering efforts by other groups can be integrated into this work, such as ontology on a geological time scale [43–46], paleoclimate data [35] and geoscience ontology works by the CGI (http://www.cgi-iugs.org/, a founding member of DDE). The knowledge system will be constructed jointly by the DDE research team and authoritative communities of experts in geoscience data standards based on existing deep-time Earth ontology studies.

The second step in DDE’s research plan is to build an interoperable deep-time Earth data infrastructure. This task will be achieved in four ways (Fig. 3): (i) harmonizing existing databases for improving interoperability and accessibility. Some important existing databases covering specific areas of Earth science may need to be extended to cover more regions (e.g. Macrostrat in sedimentology) or more languages (e.g. PBDB in paleontology and EarthChem in geochemistry). (ii) Integrating (for the ones that are not deposited in a FAIR data repository) and linking (for the ones that are deposited in a FAIR data repository) scientific data published in academic literature. With appropriate agreements and support from publishers and publisher-recommended FAIR data repositories, publications in peer-reviewed journals or books can be shared and accessed via DDE portals for information mining. (iii) Connecting databases maintained by geological surveys at all levels of government. Vast amounts of data acquired and maintained by geological surveys can be connected under DDE portals for high efficiency utilization without altering the ownership of the data. (iv) Identifying and recovering ‘dark data’, which are the data not available on the internet or, in some instances, not even in digital format. Hardcopy geological reports hibernating on shelves, thin sections of rock samples and physical samples from various geological units, or boxes of field notes from geological surveys can be digitized with the aid of modern digital techniques in collaboration with the institutions and individuals who own the property rights of the hardcopy data. One successful example of converting hardcopy materials to digital data is a collaboration between DDE and the BGS to digitize geological documents stored by BGS.

The third step in DDE’s research plan is to develop a deep-time Earth open platform. To facilitate harmonization and utilization of deep-time Earth data, a comprehensive platform is needed to provide adequate computing power, big data storage and inquiry, and big data analysis openly and freely accessible to the public. Many applications in Earth science rely on relatively small but critical data sets, owing to complications of data sampling and preservation for deep-time Earth. Specialized and tailored data analysis methods need to be developed to handle these unique deep-time data resources. Such techniques may include, but are not limited to, data visualization and interactive three-dimensional representations to elucidate planetary evolution (e.g. [47]), automation of information mining from literature [16,17], digitization and comparison of map and graphic documents for deep-time Earth, data cleaning and spatiotemporal connections for large data sets, operation and conversion of spatiotemporal references between different databases, tailored computing, and artificial intelligence methods for deep-time Earth data mining.

The execution of the DDE program consists of four phases (Table 4). In Phase 1, DDE establishes an organizational structure with international standards of policy and management. In Phase 2, DDE forms the initial teams and builds on existing deep-time Earth knowledge systems and data standards by collaborating with existing ontology researchers in the geosciences, while working to link and harmonize deep-time Earth databases. In Phase 3, DDE develops tailored algorithms and techniques for environments of cloud computing and supercomputing. In Phase 4, Earth scientists and data scientists collaborate seamlessly on compelling and integrative scientific problems.

**Table 4.** Phases of the DDE program.

	Phase 1	Phase 2	Phase 3	Phase 4
Teams	Develop team structure	Form initial teams	Research and technical teams	Mature research and technical teams
Data	Build on existing knowledge systems and data standards	Initial harmonization of databases	Continued harmonization of databases	Semantically cohesive databases
Infrastructure	No plan	Cloud storage	Cloud computing, supercomputing	Complete middle and back end
Computing and analyzing power	No plan	No plan	Develop algorithms and applications	Complete front end and Geological Google

## CHALLENGES AND COUNTERMEASURES

Due to the integrative and international ambitions of the DDE program, several challenges were anticipated. We must learn how to engage more scientists and scientific communities, how to identify and collate diverse data in many languages and formats, and how to build and share a globally integrated data infrastructure. We are also faced with the challenges of promoting international collaboration; of aligning DDE with existing and emerging data and cyberinfrastructures; and of negotiating aspects of infrastructure sustainability in the context of potential cultural and political challenges.

DDE aims to build a transparent organizational structure that attracts more scientists to join the program. This objective is reflected in the organizational structure led by international scientists, including a responsive governance structure (GC, SC, EC) and active, visionary working, platform, and task groups (program teams and open groups). In this robust organizational structure, DDE endeavors to create a vast data infrastructure with flexible data-driven discovery services, which will attract more scientists to join. Ultimately, a dynamic DDE scientific community will embrace the mission of data-driven discovery in deep-time Earth.

There are already many successful databases [12,19,32] and data infrastructures [17,35,48,49]. To avoid duplication efforts, we have reviewed all deep-time Earth databases and data infrastructures to our knowledge. We identify two kinds of databases that our efforts will leverage: first, those that lack long-term resources for maintenance and support and thus will benefit from a more stable platform; second, databases under stable development that will benefit from links to complementary data resources and infrastructure [12,19,32]. For the former, we tend to negotiate with the administrators to support them and welcome them to be part of DDE. For the latter, we plan to collaborate with them to help them grow faster, for example, by providing solutions, computing power, or taking care of multi-language and region coverage. On the other hand, DDE plans to link existing deep-time Earth databases with the resources of DDE’s knowledge graph. For the long tail data that have not been digitized or centralized, DDE will digitize and collect these data. In this regard, a number of well-established data infrastructures [17,35,48,49] provide us with valuable use cases. DDE will build our database upon theirs. For example, EarthCube is an outstanding program that is a community-driven geoscience cyberinfrastructure [49]. DataONE is an excellent model to link existing databases [48]; PaCTS 1.0 offers a solid paleoclimate ontology [35] that will be utilized as part of DDE’s deep-time Earth knowledge graph. DDE data systems and data infrastructure will follow the FAIR [4] and TRUST (Transparency, Responsibility, User focus, Sustainability and Technology) [50] principles to ensure DDE’s openness, data quality and sustainability. Furthermore, we will collaborate with CODATA and the World Data System in data policy, data standards and data management to strengthen DDE’s data system and data infrastructure.

Sustainability is another challenge to DDE. DDE is presently the prioritized long-term program in the IUGS, which secures a portion of financial support. DDE will apply for both governmental support and private support for its remaining operating expenses. DDE plans to provide seed funding for supporting members to write proposals to apply for more funding. Operating the DDE at multiple centers of excellence will mitigate risk, ensure business continuity and help disaster recovery. Each center will backup data from other centers. In addition, we plan to archive all data periodically to a vault like GitHub Arctic Vault to preserve it for future generations.

DDE may also face political challenges. To avoid this, DDE was initiated by a non-governmental organization (IUGS). DDE has instituted an agreement for anyone who joins the program to declare no political involvement. Furthermore, DDE will not accept funding that has an underlying political or military intent, nor will they strike any agreement with funding agencies that carry the risk of political influence.

## OUTLOOK

Recent advances and insights in Earth and data sciences are accelerating our ability to harness data simultaneously from many domains. The power of abductive discovery is creating opportunities to embrace the multi-dimensional complexity of Earth's evolution as never before. In so doing, we are poised for a burst of discovery that will change our perspectives about our evolving world and our place in the cosmos. In order to facilitate the transformation of deep-time Earth science, DDE plans to harmonize deep-time Earth data in linked, semantically cohesive data platforms. DDE aims to address grand challenges in the study of Earth's evolution by harmonizing deep-time Earth data, geological knowledge and modern techniques of data science and artificial intelligence. The ‘Geological Google’ and Deep-Time Earth Engine developed by DDE will facilitate Earth scientists in their efforts to find, collect and clean data, enabling them to focus on scientific questions and discoveries and address applied geological problems. DDE will also promote the integration and investigation of data in multiple dimensions, thus ushering in a new era in interdisciplinary abductive discovery in the geosciences. Ultimately, DDE will be like a virtual open restaurant, where all scientists can come and ‘cook’ using their own creativity. The data are the raw material of the cuisine. Techniques and engines developed by DDE are the cooking recipes and utensils. Customers (scientists) can come and make the food in their own way to tackle the most exciting challenges of science—discovering what we do not know, the unknown unknowns (Fig. 2). Ultimately, by creating an open-access data resource that for the first time integrates all aspects of Earth's narrated past, DDE holds the promise of understanding our planet's past, present and future in new and vivid detail.
